# Differentiation of Wharton's Jelly Derived Mesenchymal Stem Cells into Insulin Producing Cells

**Published:** 2018-07-01

**Authors:** Hossein Ranjbaran, Saeid Abediankenari, Alireza Khalilian, Zahra Rahmani, Marzei Momeninezhad Amiri, Zahra Hosseini Khah

**Affiliations:** 1 Immunogenetics Research Center, School of Medicine, Mazandaran University of Medical Sciences, Sari, Iran; 2 Department of Biostatistics and Community Medicine, School of Medicine, Mazandaran University of Medical Sciences, Sari, Iran; 3 Department of Obstetrics, School of Medicine, Mazandaran University of Medical Sciences, Sari, Iran; 4 School of Traditional Medicine, Iran University of Medical Sciences, Tehran, Iran; 5 Department of Molecular Medicine, School of Advanced Technologies in Medicine, Tehran University of Medical Sciences, Tehran, Iran

**Keywords:** Mesenchymal stem cells, Wharton’s jelly, Differentiation, Insulin producing cells

## Abstract

**Background: **Diabetes caused by insulin production disturbance is considered as the most common metabolic disorder all over the world. Diabetes may outbreak because of low insulin secretion by Islets of Langerhans β-cells, insulin resistance or both of them. In this way, using stem cells, which have the capability to differentiate into pancreatic β-cells, is one of novel methods in this field. MSCs are the most important candidates for cellular therapy.

**Materials and Methods: **Insulin level was examined using ELIZA method. In order to examine the morphology of differentiated cells, they were stained by Dithizone. Insulin-producer cells are cells which turn into red as a result of staining. Specific gene involving insulin-producing cells was evaluated by Real Time-PCR method.

**Results: **The ELISA results showed that the treated cells secreted more insulin than the control group. Moreover, we found differentiation of MSCs toward insulin-secreting cells. In order to evaluate insulin production in clusters on day 21 of differentiation, we used dithizone (DTZ) staining. PDX-1 gene was confirmed by RT- PCR analysis.

**Conclusion: **In this study, we differentiated MSCs into insulin-producing cells in vitro. It is concluded that MSCs may be considered as an excellent candidate in β-cell therapy in diabetes patients.

## Introduction

 Diabetes, caused by insulin production disturbance, is considered as the most common metabolic disorder all over the world. Diabetes may outbreak because of low insulin secretion by Islets of Langerhans β-cells, insulin resistance or both of them^1^. Currently more than 387 million people suffer from diabetes mellitus around the world. According to estimates, this figure will reach 500 million people by 2030. Generally, diabetes is divided into two types: Type I is a disorder which caused by auto immune destruction of Pancreas β-cells , and in Type II diabetes caused mainly because of progressive body resistance against insulin which results in β-cells destruction and insufficient insulin production.^2^

Although the exact source of this disorder is unknown, there are some evidence which reveal that genetic and environmental factors affect the process of auto immune excitation^3^^-^^5^. on the other hand, no conclusive treatment has been found for diabetes, while taking oral drugs and insulin injection are among common treatments. Also, Pancreas transplantation and Islets langerhans cells are also practiced. But, Pancreas transplantation is limited because of Diabetes spread and limited number of donors. 

In this way, using stem cells, which have the capability to differentiate into Pancreas β-cells, is one of novel methods in this field^6^. MSCs are the most important candidates for cellular therapy. There is fundamental indication from in vitro^7^, preclinical ^8^^-^^10^ and clinical ^11^^-^^14^ studies. Stem cells are unspecialized cells with high proliferation and have the following characteristics: regeneration, differentiation, self-renewal ^15^ if induced by a specific driver. Stem cells can divide several times and maintain multipotency, and can differentiate into specialized cells ^16^.

There are two kinds of stem cells in the bone marrow: 1) hematopoietic stem cells, and 2) Mesenchymal Stem Cells (MSCs), which differentiation into fat cells, cartilage and bone in specific circumstances^17^. Meanwhile genesis, Pancreas produces factors which cause all stem cells to differentiate into insulin-producer cells ^18^.

MSCs express surface indices MHC-I , CD25 , CD44 , CD73 , CD90 , CD105 , CD166 and is negative in terms of surface markers MHC-II , CD14,CD31 , CD34 , CD45,HLA-DR. Three main condition for approving the identity of mesenchymal are:1) in the culture, cell adherence to flask bottom, 2) expression of some markers and lacking expression of other markers, and 3) the capability to differentiate into fat, bone and cartilage cells. 

Given that providing MSCs from bone marrow is laborious, and in some cases observing ethical codes isn’t possible, researchers are looking for alternative resources, including umbilical cord blood, Wharton’s Jelly, and placenta^19^^-^^21^. Because these tissues are discarded after delivery, so they don’t put the newborn and the mother at risk. 

Researchers insist that Pancreas genesis and Neural System genesis have some similarities, although these two tissues were formed by different resources^22^^-^^24^. 

In this way, Pancreas endocrine cells are produced from embryonic stem cells using the a novel method called “neuron production” as follow:: 1) genesis of embryonic body(EB) which includes the cells differentiating the three layer 2) differentiation of cells which express nestin against fetal bovine serum reduction and culturing in ITSFN(Insulin-Transferrin-Selenium-Fibronectin), and 3) proliferation and maintaining precursor cells in the presence of alkaline fibroblastic growth factor(bFGF/FGF-2) in a medium free of serum, with N2, and B27 and 4) Induction of differentiation and maintaining positive insulin cells through adding Nicotine Amide and removing bFGF ^25^^-^^27^.

Some researchers have shown that fibroblastic growth factors can play a significant role in differentiation of pancreatic B-cells in various dilutions. In this way, many FGFs, including 22 items have been examined. These factors are secreted from various tissues and affect the target tissue^28^. 

bFGF can stimulate angiogenesis in vitro and in vivo, and help diabetes treatment by angiogenesis. FGF10 play its role in signaling mesenchymal- epithelial interaction and cell proliferation. 

Other researchers have argued that the presence of neuro-epithelial protein nestin in human and rat islets Langerhans may by an indicator for endocrine precursor cells. Also, they showed that nestin is present in endothelial and mesenchymal cells of vessels inside and outside the islets of Langerhans, and showed that the cells proliferated in mature islets Langerhans are primarily endocrine^29^^-^^32^.

Wang and Phuc et al. (2011) could create pancreatic β-cells from mesenchymal stem cells of umbilical cord blood, which, after injection, could lower blood glucose of diabetic rat. 

They found that mesenchymal stem cells of umbilical cord vein wall have less capacity in terms of differentiation, compared to Wharton’s Jelly mesenchymal cells and umbilical cord blood. Lower differentiation capacity is obvious in bone and fat differentiation^33^^,^^34^.

Another research (2010) showed that growth factors, including retinoic acid, FGF and activin are used for differentiation of stem cells into cells which express PDX-1^35^.

Until now, many researches have tried to produce mesenchymal stem cells and their differentiation into pancreatic β-cells using various molecules; unfortunately, they couldn’t produce prefect pancreatic β- cells. 

This research aims at isolation mesenchymal stem cells from Wharton’s Jelly. The differentiation of MSCs to pancreatic cells was evaluated in presence of ActivinA, Retinoic Acid, Fibroblast Growth Factor10, Nestin and Forskolin in culture medium. The concentration of the components, mentioned above, was also optimized in the culture. Thus, a novel method for producing pancreatic cells may be explained. 

## MATERIALS AND METHODS

**Collection of human umbilical cord**

In an experimental study, human umbilical cord samples were collected in fully sterilized condition from six mothers after delivery at the Obstetrical Department of Imam Educational and Therapeutic Hospital, Sari, North of Iran. Then samples were transported to the Medical University. 

**Preparation and culture of Wharton’s jelly**

First, in a 10 mm-plate, all clots and arteries of umbilical cord were isolated under laminar hood. Then, the gelatinous substance within the umbilical cord, Wharton jelly, was cut into small pieces mechanically, and was rinsed several times with Phosphorous buffered saline (PBS) at 1250 rpm for 5 min. For lysing the remaining RBC, 2 ml Hypertonic Chloride, Ammonium (USA, Pharmin Gen) was added to the sample and was rinsed after 10 min. Cell deposition, which included cells that separated from the placenta tissue, was transferred to T75 Flask and was cultured in High Glucose-Dulbecco's Modified Eagle Medium- F12 (HG- DMEM-F12) (USA, Gibco) supplemented with 10 % fetal bovine serum (FBS) and 50 u/ml Penicillin-Streptomycin. The plate was incubated in 5% CO2 at 37 ºC. 

**Isolation and characterization of Mesenchymal Stem Cells (MSCs) from other umbilical cord cells**

The medium was changed after 24 hours; the suspended particles (non-mesenchymal cells) migrated from the medium and the mesenchymal cells adhered to flask, and turned into spindle-like shapes. When the confluency of the cells reached 80-90%, the mesenchymal cells were harvested using 0.25% trypsine including 1ml/M EDTA (USA, Sigma). 

The suspension was centrifuged at 1250 rpm for 5 min. The cell deposition was rinsed two times and centrifuged. Finally, it was filtered using grade 70 micron filter. 

To approve MSCs identity, positive markers of stem cell surface such as CD105, CD90, CD44 and negative markers, CD34 and HLA-DR were evaluated by flow cytometry. Conjugating related antibodies, Fluor chromes FITC, PE and PerCP, isotype controls of PerCP, PE and IgG1-FITC were used. 

The WJ-MSCs were induced to differentiate into adipocytes, osteocytes and chondrocytes. The differentiation protocol has been described previously^26^. In this way, WJ-MSCs at 1.6 × 10^5^ cells/ml were cultured in HG-DMEM-F12 complemented with 15% FBS and 2mMol/ L- glutamine and Penicillin-Streptomycin for 6 days. The cells were then fixed with 0.4% paraformaldehyde (PFA) and stained with oil-red-o (sigma) to confirm the adipocyte. For osteoblastic differentiation, the WJ-MSCs were cultured in HG-DMEM-F12 complemented with 10 nMol/L b-glycerol phosphate (sigma) and 50mg/ml ascorbic acid-2 phosphate (sigma). After fixation, the differentiated cells were stained with Alizarin red, and then the calcium deposition was confirmed. For chondrogenesis differentiation, WJ-MSCs were seeded in a HG-DMEM-F12 complete culture media at 1.6 × 10^5^ cells/ml with 5 μl of chondrogenesis culture media for 14 days. The micromass cultures were then stained with Alcian blue.

**Differentiation of MSCs into insulin-producing cells**

After isolation and confirmation of MSCs, optimization of Activin A, Retinoic Acid, Fibroblast Growth Factor10, Nestin and Forskolin in various dilutions were checked in the culture, and the optimum dilution was obtained. Then induction treatment was conducted in 3-5 days. Stimulating cell proliferation, various additives, including (Gibco) B27 and (Gibco) N2 were added to the culture. 

**Dithizone staining**

To examine the morphology of differentiated cells, they were stained with Dithizone by completely dissolving 50 mg of DTZ (Sigma) in 5 ml of dimethyl sulfoxide (DMSO, Sigma). The solution was stored in -20 ᵒC in the dark. At the time of staining, the working solution was prepared by diluting the stock solution (pH 7.8) to a ratio of 1:100 in the culture medium. We added 3 ml of the working DTZ solution to each well and the plates were incubated for 30 min at 37ᵒC. The plates were then rinsed three times with PBS and crimson-red differentiated clusters were examined with an inverted phase contrast microscope.

**Enzyme-linked immunosorbent assay (ELISA)**

For evaluation of insulin secretion, the supernatant of differentiated cells was collected and insulin content was examined by ELISA method using an Insulin ELISA kit (Demeditec, Germany) according to the protocol.

The evaluation of the PDX-1 expression in mRNA level which confirms the insulin-producing cells was done by reveres transcription polymerase chain reaction (RT-PCR).

**RNA extraction and cDNA synthesis**

Extraction of total RNA from differentiated cells was used by Accuzol^TM^, a ready-to-use reagent (Bioneer, Korea) according to the manufacturer’s protocol. After evaluating the quality and quantity of RNA by electrophoresis and spectrophotometer, respectively, cDNA synthesis was done by AccuPower® CycleScript RT Premix (dN6) (Bioneer, Korea). The concentration of RNA used for cDNA synthesis was 1µg. The thermal profile for cDNA synthesis was: primer annealing at 25℃ for 30 s, cDNA synthesis at 45 ℃ for 4 min and melting secondary structure & cDNA synthesis at 55℃ for 30 s and repeated 12 times as well as heat inactivation at 95℃ for 5 min.

**Reverse transcription polymerase chain reaction (RT-PCR)**

For assessment of insulin-producing cells, we evaluated expression of PDX-1 gene, which was related to insulin production. First, to set the primers, we performed conventional PCR. PCR amplification was done for PDX-1 gene and EF-1 as reference gene, in a Thermocycler (Eppendorf, UK) by using 2μl of CDNA, 1μM of each primer, and 12.5μl of Taq DNA Polymerase 2x Master Mix, Red (Ampliqon, Denmark) in a 20 µl total volume reaction. 

The primer sequences were as follows: EF1 (Forward): 5´-CTGAACCATCCAGGCCAAAT-3´ and EF1 (Reverse): 5´-GCCGTGTGGCAATCCAAT-3´ which amplified a 59 bp fragment and for PDX1 gene we used GGATGAAGTCTACCAAAGC-3´ as a forward and 5´-CGTGAGATGTACTTGTTGAA-3´ as reverse to amplify a 157 bp fragment. The thermal profile for amplification were 95ᵒC for 2 min for initial denaturation, followed by 40 cycles denaturation at 95ᵒC for 30s, annealing at 45ᵒC for EF-1 and 56ᵒC for PDX-1 for 30s, extension at 72ᵒC for 30S, and final extension at 72ᵒC for 2 min. 

PCR products were electrophoresed on 2% agarose gel. Electrophoresis was performed at 100v for 40 min. Fragments with 59 bp and 157 bp were scored as EF1 and PDX1 gene, respectively. 

**Real-time polymerase chain reaction (RT-PCR)**

Evaluation of PDX1 expression at mRNA level was performed using the real-time PCR system (IQ5, Biorad, USA). The EF1 gene was used as an endogenous reference gene allowing normalization of the expression level of the target gene to normalize the expression levels of target gene. Primer sequence was mentioned in RT-PCR part. We used SYBR Premix EX TaqII (2X) (Takara, Cat. #: RR820L) master mix for amplification. The thermal profile for PDX-1 and EF-1 were as follows: The thermal profile was as follows: Initial denaturation at 95°C for 2 min, followed by 45 cycles of denaturation at 95°C for 30 s, primer annealing for PDX-1 at 56°C and for EF-1 at 45°C for 30 s, and extension at 72°C for 30 s. This cycle was followed by a melting curve analysis, ranging from 45°C for EF-1 and 56°C for PDX-1 to 95°C, with increasing steps in temperature ( 0.5°C every 10 s). 

**Statistical analysis**

**Figure 6:**
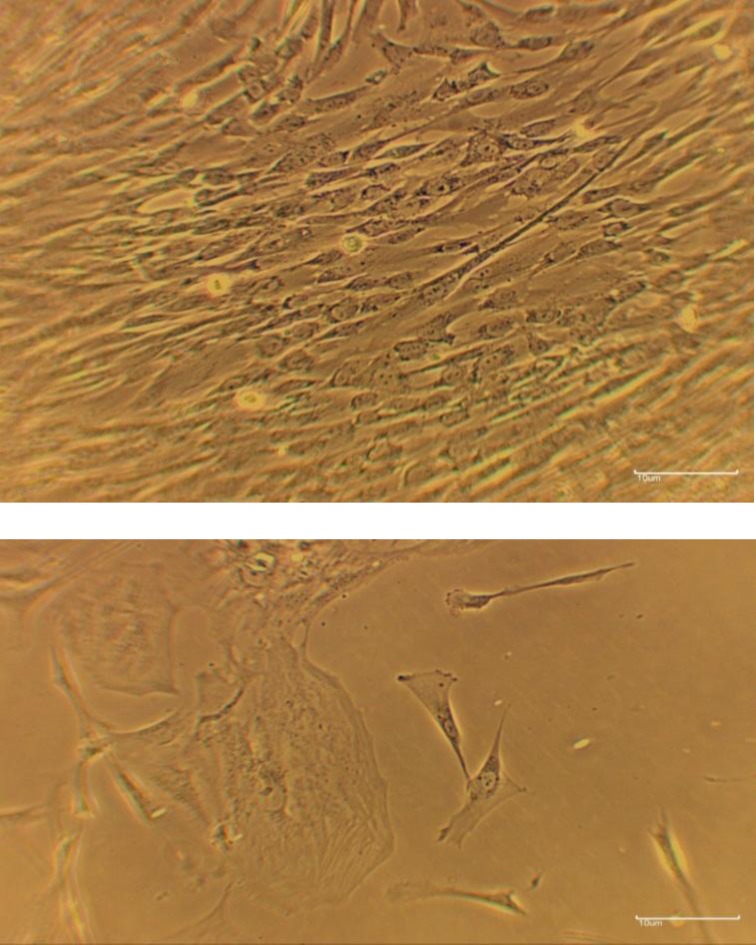
PD X-1 gene expression in MSCs and IPCs P-value = 0.001

The relative expression of PDX-1 was calculated by 2^-ΔΔCt^, as previously described by Livak^36^.The data were presented as means ± SD. Data were assessed by one-way ANOVA followed by Tukey’s test for comparison of the experimental group. The statistical analysis was performed using SAS software. P-values <0.05 were considered statistically significant (Figure 6).

## Results

 In our study, we differentiated Wharton jelly´s mesenchymal stem cells (WJ-MSCs) toward insulin-producing cells (IPCs). For this purpose, after isolation and differentiation of WJ-MSCs toward IPCs, we performed the morphological and molecular analysis. 

**Morphological and Phenotypical Characterization of WJ-MSCs**

**Figure1:**
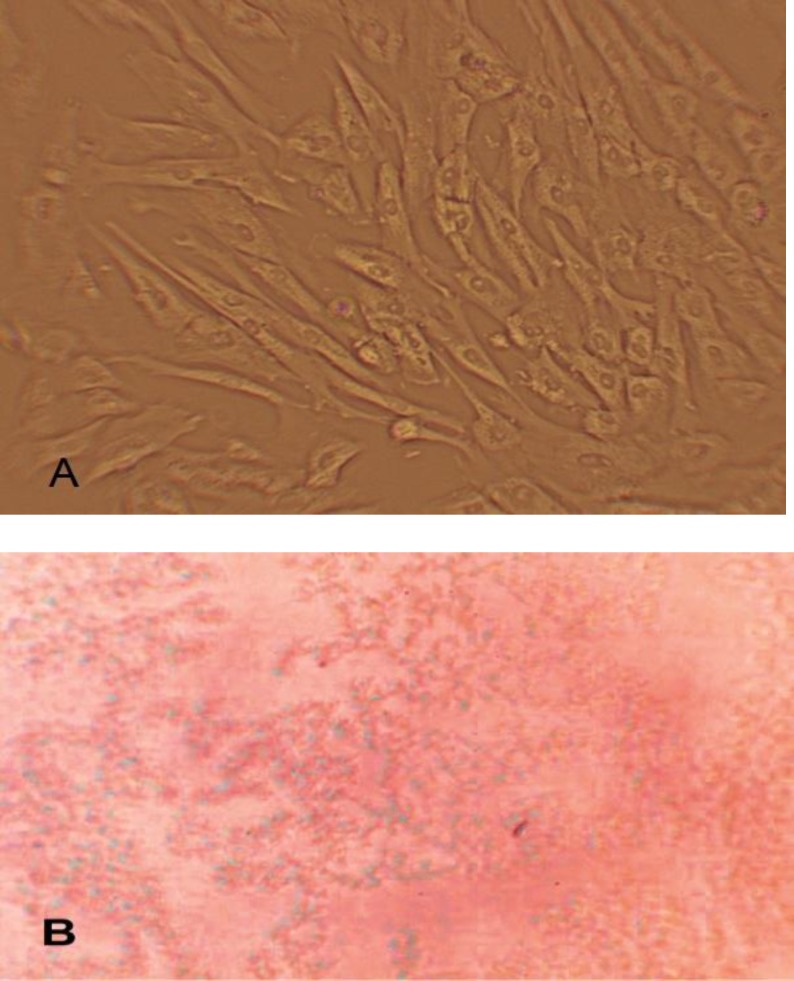
The Wharton`s Jelly mesenchymal cells were grown from

At the end of the expansion phase, the cultured WJ-MSCs become homogenous, spindle shaped, and fibroblast-like to arrange in monolayers (Figure 1)*.*

Flow cytometric analysis showed that these cells expressed high levels of CD90, CD105 and CD44, but negligible levels of CD34, and HLA-DR, which are surface markers for hematopoietic stem cells. Moreover, their multilineage differentiation potential was confirmed. The cells have the potential to differentiate into adipocytes, chondrocytes, and osteocytes when they were exposed the appropriate growth factors. These data indicate that MSCs were the majority of the WJ-derived cells. 

**Dithizone (DTZ) staining**

**Figure2:**
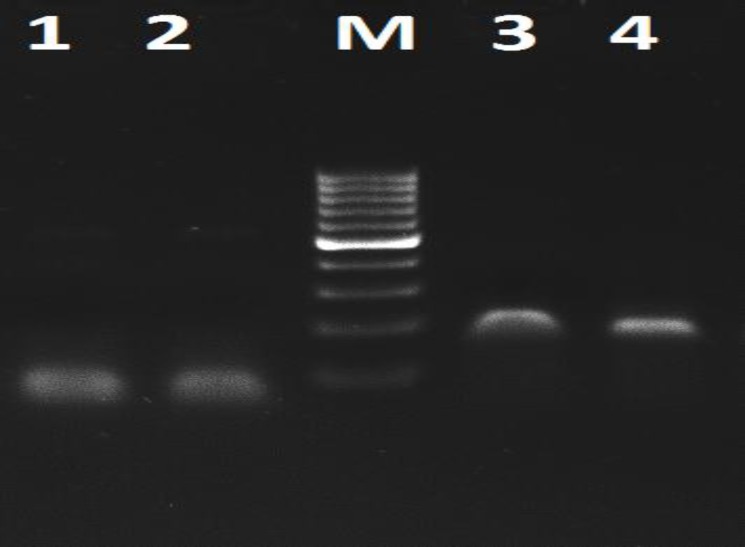
DTZ (Dithizone) staining; observed with inverted microscope and magnified ×100 A: Untreated cells B: In cells treated with dithizone, insulin granules come in red brick.

In order to evaluate insulin production in clusters at day 21 of differentiation, we used dithizone (DTZ) staining. The stock solution was prepared as previously described^37^. Insulin-producing cells are the cells which turn into red in result of staining (Figure 2).

**ELISA test**

In order to determine the amount of insulin secreted by the differentiated cells after the day 28, the cell culture medium was tested by using ELISA technique. The results showed that the differentiated cells compared to the undifferentiated cells, secrete insulin. We could be able to differentiate MSCs toward the insulin producing cells.

**PDX-1 expression in IPCs**

**Figure 3:**
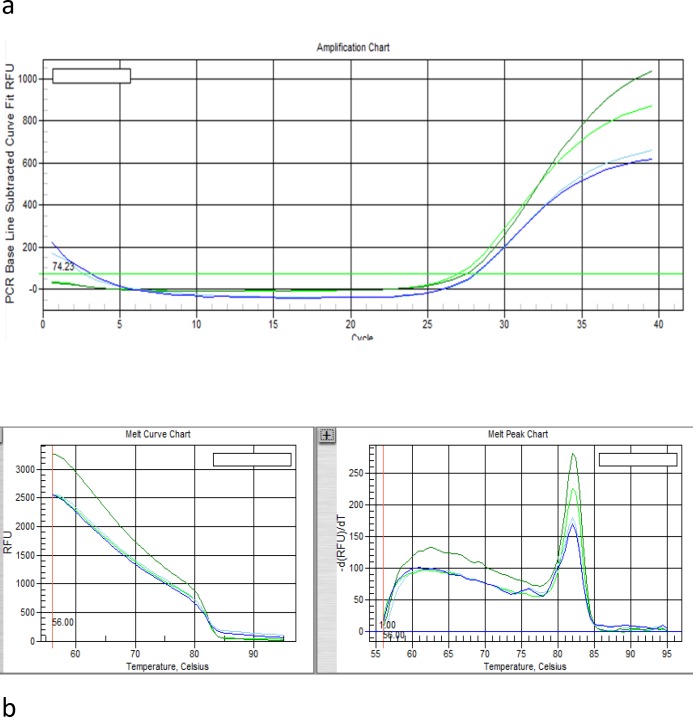
Electrophoresis of PDX-1 and EF-1 on 2% agarose gel; M shows marker (100bp); Lane 1, 2 represent PCR product of the EF-1 amplification in 45ᵒC; Lane 3, 4 represent PCR product of the PDX-1 amplification in 56ᵒC

**Figure 4:**
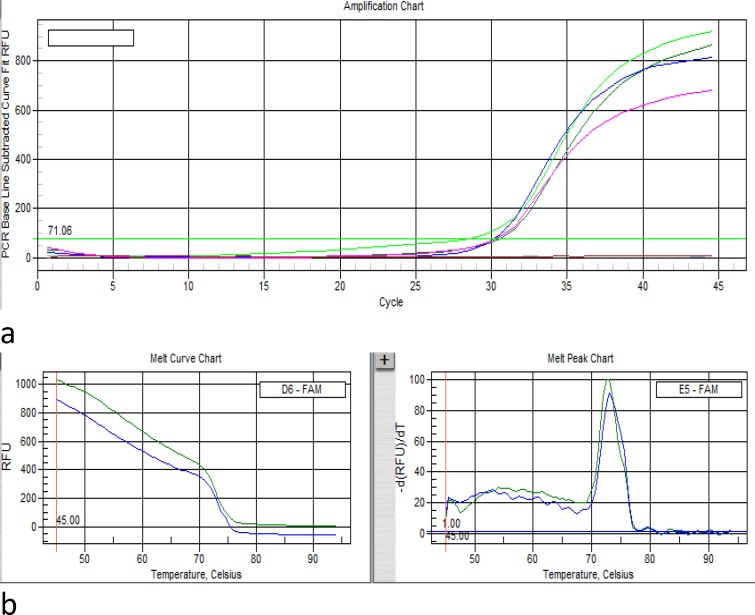
Amplification plot (a) and melt curve (b) of EF1- Reference Gene

**Figure5:**
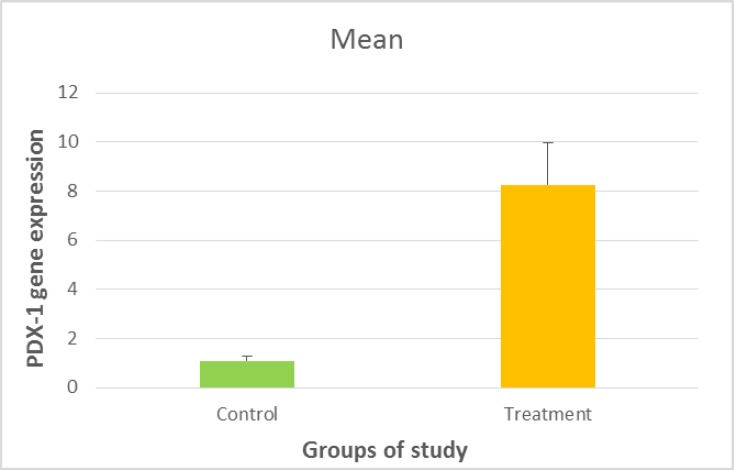
Amplification plot (a) and melt curve (b) of PDX-1Gene

After differentiation, the expression of β-cell specific gene such as PDX-1 was assessed in Wharton jelly mesenchymal stem cell and insulin-producing cells by qRT-PCR. The results of RT-PCR for primers assessment are shown in Figure3. The amplification plot and melt curve of PDX-1 and EF-1 represent in Figures 4-a,b and 5-a,b respectively. The expression levels of PDX1 versus EF1 in IPCs were7.55 fold-changes higher than WJ-MSCs, and it was statistically significant (p= 0.001) (Figure 5).

## Discussion

 MSCs are self-renewing cells with multilineage differentiation potential^38^. The ease of isolation and expansion has rendered any potentially significant source of stem cells as it could be used in the tissue engineering and regenerative medicine, whereas it represents a therapeutic potential for DM^33^. Previously, some publications demonstrated the differentiation capability of adult stem cells toward the pancreatic cell lineage^39^^-^^43^. Recent studies have revealed the potential of MSCs from different sources for the treatment of the diabetes mellitus ^44^^,^^45^ and the diabetic complications, including diabetic cardiomyopathy^46^, diabetic retinopathy^47^, diabetic polyneuropathy^48^, diabetic nephropathy^49^ and diabetic wounds^50^. MSCs can be isolated from several organs and tissues, e.g. bone marrow, dental pulp, adipose tissues, and umbilical cords^51^. Wharton`s Jelly method was used to derive MSCs, by which several significant advantages have been reflected such as the high number and the relatively easy isolation^52^. MSCs are capable to differentiate into islet-like cells and it has immunomodulatory abilities subject to the possibility to reduce the risk of immune rejection^53^. Insulin Producing Cells (IPCs) can be obtained by two methods: indirect and direct differentiation. The indirect differentiation application for the chemicals, for example, nicotinamide and growth factors are shown as inductors. In this method, the application of the high glucose concentration in the medium is critical, since it is a potent inducer of differentiation. Another method is direct differentiation, which is based on the modification of the genetic material, for example, by using the viral vectors^54^.

Raikwar et al. (2013) reported the ectopic expression of Pancreatic and duodenal home box Factor-1(PDX-1) which is a critical transcription factor for pancreas, and increased embryonic stem cells differentiation into insulin producing cells. 

Sipione and Hanson and Rajagopal et al. (2003-2004) tried to show that transplanted stem cells promote treatment of diabetes. They, in their research, reported that neurons and neural precursors are the main producers of insulin/proinsulin ^55^^-^^57^.

Moreover, in a study which yielded 7.3u/ml insulin, endodermal layer cells were sustained using Activin A and then adding FGF10 and retinoic acid led to PDX-1 cells expression as the main marker of the insulin producing cells^58^^-^^59^. 

In other study, researchers showed that endodermal cells can generate pancreatic precursors in exposure of Retinoic acid and cyclopamin^60^^, ^^61^. 

One of the important signs of the beta pancreatic cells is the insulin secretion. Thereby, in the present study, the secretion rate of insulin in the differentiated MSCs was studied.

In this research, after the successful isolation of mesenchymal stem cells (MSCs) and its confirmation via three approaches, including adhesion of fibroblast spindle-shaped cells at the bottom of the flask and the differentiation into the fat, bone and cartilage cell lines and the examination of the positive and negative CD markers, in the next phase, we conducted the differentiation into the pancreatic β cells with Activin A (100 ng/ml), Retinoic Acid (0.2 nM/ml), Fibroblast Growth Factor10 (20 ng/ml), Nestin (50 ng/ml) and Forskolin (250 ng/ml) and optimized their concentration in the various dilutions in the culture medium, where we could produce the insulin producing cells. 

In this study, mesenchymal stem cells (MSCs) were differentiated into the pancreatic β cells with Activin A, Retinoic Acid, Fibroblast Growth Factor10, Nestin and Forskolin and optimized their concentration in the various dilutions in the culture medium, where we could produce adequate the insulin secreting cells. In our study, the best molecular combination to produce insulin was Forskolin+Activin A+ Fibroblast Growth Factor10 and Retinoic Acid (211/7u/ml), as it was determined by ELISA.

It is required to study the combination of the differentiate factors mentioned above in the animal model. In respect to the efficient role of the in vivo compared to the in vitro on the differentiation, the study of this subject seems indispensable.

## CONCLUSION

 Mesenchymal stem cells were differentiated into the pancreatic β cells with Activin A, Retinoic Acid, Fibroblast Growth Factor10, Nestin and Forskolin and optimized their concentration in the various dilutions in the culture medium. We optimized the best molecular combination to produce adequate insulin level. Therefore, it is concluded that MSCs may be considered as an excellent candidate in β- cell therapy in diabetes patients.
